# Genome Sequence of *Vibrio cholerae* G4222, a South African Clinical Isolate

**DOI:** 10.1128/genomeA.00040-13

**Published:** 2013-03-14

**Authors:** Wouter J. le Roux, Wai Yin Chan, Pieter De Maayer, Stephanus N. Venter

**Affiliations:** a Natural Resources and the Environment, CSIR, Pretoria, South Africa; b Forestry and Agriculture Biotechnology Institute and Department of Microbiology and Plant Pathology, University of Pretoria, South Africa; c Center for Microbial Ecology and Genomics, Department of Genetics, University of Pretoria, South Africa

## Abstract

*Vibrio cholerae*, a Gram-negative pathogen autochthonous to the aquatic environment, is the causative agent of cholera. Here, we report the complete genome sequence of *V. cholerae* G4222, a clinical isolate from South Africa.

## GENOME ANNOUNCEMENT

*Vibrio cholerae* is the causative agent of cholera, a severe diarrheal disease that remains a socioeconomic burden in many developing countries (1). As such, the species, also widely regarded as a model organism for studies pertaining to water-borne pathogens, has received much attention (2). Among other foci, emphasis has been placed on defining serotypes and biotypes in an attempt to understand this rapidly evolving pathogen (3). However, with the discoveries of intraspecies serogroup transfer and contradicting biotype-related phenotypes, the focus has shifted away from classic diagnostic markers to genome-based comparisons with an emphasis on mobile elements (3, 4). To this end, the genome sequence of *V. cholerae* G4222, a clinical O1 isolate obtained in South Africa during the 2000-2001 epidemic, was determined. This represents the first genome of a South African *V. cholerae* strain. In light of the dearth of African *V. cholerae* strain sequences and the recent discovery of new recombinant *V. cholerae* biotypes in southern Africa, this genome might provide valuable insights into the evolution of this pathogen (5, 6). Furthermore, the genome sequence of this strain may contribute to the understanding of the *V. cholerae* mobilome.

The genome was sequenced using a Roche 454 GS-FLX sequencer at Inqaba Biotec, South Africa. A total of 201,286 reads with an average read length of 236 bp were obtained, giving a total of 47,503,496 nucleotides and genome coverage of 11.6×. The reads were assembled into 280 contigs using Newbler assembler v2.6 (454 Life Sciences). These contigs were then scaffolded by alignment against the complete genome sequences of *V. cholerae* MJ-1236 (7) and *V. cholerae* O1 biovar El Tor strain N16961 (8) with the NCBI Genomic (NG) Aligner tool of the NCBI Genome Workbench v2.5.5. A further 38 gaps were closed by PCR amplification and Sanger sequencing. This resulted in the assembly of the *V. cholerae* G4222 genome sequence into a total of 21 contigs. Protein-coding sequence (CDS) prediction and functional annotation of the predicted proteins were done using the Rapid Annotations using Subsystems Technology (RAST) Web server (9) before manual curation was performed.

The *V. cholerae* G4222 contigs could be scaffolded into two distinct chromosomes, as is typical of *V. cholerae* strains (10). Chromosome I consists of 14 contigs amounting to a total length of 3,139,654 bp, with an average G+C content of 47.72% and 2,809 annotated CDS. Chromosome II consists of seven contigs with a total length of 1,061,058 bp and a G+C content of 46.88%, with 1,051 CDS annotated. The chromosome sizes and G+C compositions correlate well with those of other *V. cholerae* strains (6, 8). An ~150-kb integrative conjugative element (ICE), belonging to the SXT family, is located on chromosome I of *V. cholerae* G4222 and carries the genes involved in multiple-drug resistance. Given that an African origin for SXT-related ICEs in *V. cholerae* strains has been proposed, the genome of *V. cholerae* G4222 provides further opportunity to investigate the evolution of SXT elements (11). The strain might also provide insights into the biology of South African epidemic *V. cholerae* strains.

### Nucleotide sequence accession numbers.

This Whole Genome Shotgun project has been deposited at DDBJ/EMBL/GenBank under the accession no. ANNB00000000. The version described in this paper is the first version, ANNB01000000.

## References

[1] World Health Organization (WHO) . 2012 Cholera, 2011. Wkly. Epidemiol. Rec. 87(31/32):289–304 http://www.who.int/wer/2012/wer873132/en/22905370

[2] Yildiz F 2007 *Vibrio cholerae*: model organism to study bacterial pathogenesis - interview. J. Vis. Exp. 4:e207 10.3791/207 PMC2556167 18979011

[3] Cho YJ Yi H Lee JH Kim DW Chun J 2010 Genomic evolution of *Vibrio cholerae*. Curr. Opin. Microbiol. 13:646–651 20851041 10.1016/j.mib.2010.08.007

[4] Safa A Nair GB Kong RY 2010 Evolution of new variants of *Vibrio cholerae* O1. Trends Microbiol. 18:46–54 19942436 10.1016/j.tim.2009.10.003

[5] Faruque SM Tam VC Chowdhury N Diraphat P Dziejman M Heidelberg JF Clemens JD Mekalanos JJ Nair GB 2007 Genomic analysis of the Mozambique strain of *Vibrio cholerae* O1 reveals the origin of el Tor strains carrying classical CTX prophage. Proc. Natl. Acad. Sci. U. S. A. 104:5151–5156 17360342 10.1073/pnas.0700365104 PMC1829278

[6] Grim CJ Hasan NA Taviani E Haley B Chun J Brettin TS Bruce DC Detter JC Han CS Chertkov O Challacombe J Huq A Nair GB Colwell RR 2010 Genome sequence of hybrid *Vibrio cholerae* O1 MJ-1236, B-33, and CIRS101 and comparative genomics with *V. cholerae*. J. Bacteriol. 192:3524–3533 20348258 10.1128/JB.00040-10 PMC2897672

[7] Chun J Grim CJ Hasan NA Lee JH Choi SY Haley BJ Taviani E Jeon YS Kim DW Lee JH Brettin TS Bruce DC Challacombe JF Detter JC Han CS Munk AC Chertkov O Meincke L Saunders E Walters RA Huq A Nair GB Colwell RR 2009 Comparative genomics reveals mechanism for short-term and long-term clonal transitions in pandemic *Vibrio cholerae*. Proc. Natl. Acad. Sci. U. S. A. 106:15442–15447 19720995 10.1073/pnas.0907787106 PMC2741270

[8] Heidelberg JF Eisen JA Nelson WC Clayton RA Gwinn ML Dodson RJ Haft DH Hickey EK Peterson JD Umayam L Gill SR Nelson KE Read TD Tettelin H Richardson D Ermolaeva MD Vamathevan J Bass S Qin H Dragoi I Sellers P McDonald L Utterback T Fleishmann RD Nierman WC White O Salzberg SL Smith HO Colwell RR Mekalanos JJ Venter JC Fraser CM 2000 DNA sequence of both chromosomes of the cholera pathogen *Vibrio cholerae*. Nature 406:477–483 10952301 10.1038/35020000 PMC8288016

[9] Aziz RK Bartels D Best AA DeJongh M Disz T Edwards RA Formsma K Gerdes S Glass EM Kubal M Meyer F Olsen GJ Olson R Osterman AL Overbeek RA McNeil LK Paarmann D Paczian T Parrello B Pusch GD Reich C Stevens R Vassieva O Vonstein V Wilke A Zagnitko O 2008 The RAST server: rapid annotations using subsystems technology. BMC Genomics 9:75 18261238 10.1186/1471-2164-9-75 PMC2265698

[10] Trucksis M Michalski J Deng YK Kaper JB 1998 The *Vibrio cholerae* genome contains two unique circular chromosomes. Proc. Natl. Acad. Sci. U. S. A. 95:14464–14469 9826723 10.1073/pnas.95.24.14464 PMC24396

[11] Burrus V Marrero J Waldor MK 2006 The current ICE age: biology and evolution of SXT-related integrating conjugative elements. Plasmid 55:173–183 16530834 10.1016/j.plasmid.2006.01.001

